# 

**Published:** 1885-04

**Authors:** 


					﻿io efts, a coj»y.
APRIL, 1885.
$1.00 per Year.
HAI.E5«
.pA KWI ■
HEALTH
ESTABLISHED 1854
Vi 3 2
c/Ve.- 4-
Office, 75 & 77 Barclay Street, New York.
				

